# Brain Infection by Hepatitis E Virus Probably via Damage of the Blood-Brain Barrier Due to Alterations of Tight Junction Proteins

**DOI:** 10.3389/fcimb.2019.00052

**Published:** 2019-03-19

**Authors:** Jijing Tian, Ruihan Shi, Tianlong Liu, Ruiping She, Qiaoxing Wu, Junqing An, Wenzhuo Hao, Majid Hussain Soomro

**Affiliations:** ^1^Laboratory of Animal Pathology and Public Health, Key Laboratory of Zoonosis of Ministry of Agriculture, College of Veterinary Medicine, China Agricultural University, Beijing, China; ^2^Institute of Animal Husbandry and Veterinary Medicine, Beijing Academy of Agriculture and Forestry Sciences, Beijing, China

**Keywords:** Hepatitis E virus, pathological changes, blood-brain barrier, tight junction, human brain microvascular endothelial cells

## Abstract

Extrahepatic injury, particularly neurologic dysfunctions such as Guillain-Barré syndrome, neurologic amyotrophy, and encephalitis/meningoencephalitis/myositis were associated with HEV infection, which was supported by both clinical and laboratory studies. Thus, it is crucial to figure out how the virus invades into the central nervous system (CNS). In this study, CNS lesions were determined in rabbits and Mongolian gerbils inoculated with genotype 4 HEV. Junctional proteins were detected in HEV infected primary human brain microvascular cells (HBMVCs). Viral encephalitis associated perivascular cuffs of lymphocytes and microglial nodules were observed in HEV infected rabbits. Both positive- and negative-strand of HEV RNA was detected in brain and spinal cord in rabbits intraperitoneally infected with HEV at 28 dpi (days postinoculation), but not in rabbits gavaged with HEV. HEV ORF2 protein was further examined in both brain and spinal cord sections of infected rabbits, with positive signals located mainly in neural cells and perivascular areas. Ultrastructural study showed thickened and reduplicated basement membranes of capillary endothelium in HEV RNA positive brain tissues. *In vitro* study showed loss of tight junction proteins including Claudin5, Occludin, and ZO-1 (zonula occludens-1) in HBMVCs inoculated with HEV for 48 h. These findings indicated that disruption of the blood-brain barrier (BBB) might be potential mechanisms of HEV invasion into the CNS. It provides new insights to further study HEV associated neurologic disorders and will be helpful for seeking potential therapeutics for HEV infection in the future.

## Introduction

Hepatitis E virus (HEV) infection is a global public health problem. HEV is the leading cause of acute hepatitis in the world, which is responsible for 20 million infections and 70,000 HEV-related deaths annually (Montpellier et al., 2018). There are 4 genotypes of HEV, while only genotype 3 and 4 which are zoonotic, were reported in both domestic animals such as swine and human beings. We have reported infection of HEV in Himalayan Griffons foodborne, suggesting a interspecies transmission of HEV (Li H. et al., 2015). Foodborne transmission of HEV between animals and human through preparing or eating animal products was also reported in many countries (Tei et al., 2003; Li et al., 2005; Renou et al., 2014b). HEV can also be transmitted by blood donation or liver transplantation (Dalton and Izopet, 2018). Moreover, Watercourses contaminated by HEV-infected animals or animal products, either wild or domestic animals involved in agricultural practices, can be potential sources of infectious HEV (Doceul et al., 2016; Li et al., 2017). A recent study showed that contacting with pets and farm animals resulted in a high frequency of HEV infection (Abravanel et al., 2018). Thus, food and water contamination by HEV as well as directly contacting with HEV infected animals might be the most potential risks of HEV infection between animals and human beings.

To date, multiple experimental models of HEV were established, including animal models and cell culture systems. Animals, especially non-human primate species including chimpanzee, owl monkey, squirrel monkeys, and rhesus macaques were proved to be susceptible to HEV (Vitral et al., 1998). HEV infection in swine and other laboratory animals such as rabbits and *Mongolian Gerbils* provided useful tools in further understanding HEV-associated injuries (Li et al., 2009; Mao et al., 2014; Yang et al., 2015; Dalton and Izopet, 2018). Recent studies showed that several cell lines or primary cells can be used for modeling HEV infection, including human liver cell lines PLC.PRF/5, Huh-7, and HepG2/C3A, human lung epithelial cell line A549, mouse embryonic fibroblasts, and various human neural cells lines induced pluripotent stem cell-derived human neurons and primary mouse neurons (Zhang and Wang, 2016; Zhou et al., 2017). Both laboratory animals and cell cultural systems are helpful to further explain mechanisms underlying HEV infections, to develop vaccines and therapeutics for the prevention and treatment of HEV infections.

In recent years, extrahepatic injury of HEV, including renal diseases, reproductive system disorders, as well as pancreatitis, and a variety of neurological disorders after HEV infection, were documented (Soomro et al., 2016, 2017; Dalton et al., 2017; Pischke et al., 2017). Neurological syndromes of HEV infection, including neuralgic amyotrophy, cerebral ischemia or infarction, seizure, encephalitis and acute combined facial, and vestibular neuropathy, were observed in a clinical cohort study in patients with non-traumatic neurological illnesses (Dalton et al., 2017). A clinical cohort study in France showed that 137 out of 200 HEV-infected patients (16.5%) suffered from neurological manifestations (Abravanel et al., 2018), highlighting that the central nervous system (CNS) are targets of HEV infection. Another study showed that HEV RNA and viral protein ORF2 could be detected in brain tissues of mice and monkeys infected with HEV experimentally (Zhou et al., 2017). We have shown that various pathological changes of the CNS in Mongolian gerbils were correlated with HEV infection, such as perineural invasion, neuron necrosis, microglia nodule, lymphocyte infiltration, perivascular cuff and myelin degeneration. The blood-brain barrier (BBB) associated proteins such as ZO-1 (zonula occludens-1) and GFAP (glial fibrillary acidic protein) were further confirmed dysfunction in HEV infected animals (Shi et al., 2016). However, how the virus transmitted into the CNS and lead tissue damages are still not clear. The aim of the present study was to investigate pathological changes of the CNS in rabbits experimentally infected with HEV and roles of junctional complex proteins such as Claudin5, Occludin, ZO-1, and VE-cadherin in primary human brain microvascular endothelial cells (HBMVECs) during HEV infection.

## Materials and Methods

### Virus, Inoculation, Animals, and Sampling

The HEV strain used for inoculation was genotype 4 swine HEV, which was derived from a swine intestinal content (GenBank accession number KJ123761) (Wu et al., 2017). Briefly, infectious virus stock was generated from the fecal samples of 2 rabbits inoculated with the intestinal content suspension intraperitoneally, with a titer of 6.63 × 10^7^ copies per ml and stored at −80°C prior to inoculation.

Thirty two 80-day-old female New Zealand white rabbits weighing between 1,800 and 2,000 g were purchased from the Xing Long Experimental Animal Center, Beijing, China. The blood, feces and serum of all the rabbits were confirmed to be negative for HEV RNA or HEV antigen and antibodies before inoculation. Each rabbit in the experimental groups was inoculated with 10 mL of prepared viral homogenate per day via intraperitoneal injection or gavage for 7 consecutive days. Rabbits inoculated with the same dose of HEV-negative intestinal homogenate served as control group. Each rabbit was housed in a separate cage, and monitored every day. No clinical symptoms were observed in the experimental rabbits. The detailed protocol was described as Wu et al. (2017).

Rabbits were sacrificed at 7, 28, and 49 days post-inoculation (dpi) of the intraperitoneal injection group (labeled with 7A−7B, 28A−28D, 49A−49D), and 49 and 63 dpi of the gavaged rabbits (labeled with 49A–C, 63A–C), respectively. Brain and spinal cord of the rabbits were collected and fixed in 2.5% (v/v) glutaraldehyde-polyoxymethylene solution, or stored at −80°C immediately for viral RNA extraction. All animal protocols were approved by the Animal Care and Use Committee of China Agricultural University and were performed humanely for alleviation of suffering.

The HEV strain used for Mongolian gerbils inoculation was genotype 4 swine HEV, derived from a swine liver sample (CHN-HB-HD-L2, GenBank accession number KM024042). Briefly, animals were inoculated with the HEV virus and sacrificed at 0, 7, 14, 21, 28, 42, and 56 days dpi. Brain and spinal cord tissues were collected and fixed in 2.5% (v/v) glutaraldehyde-polyoxymethylene solution for further examination. The detailed information was described as Shi et al. (2016).

### RNA Extraction and PCR Protocols

Total RNA was extracted from brain and spinal cord tissues with the Ultrapure RNA kit (CWBIO, Beijing, China) and reverse transcription were performed using HiFi-MMLV kit (CWBIO, Beijing, China) according to the instructions of the manufacturer. RTnPCR-positive brain and spinal cord tissues were arranged for real-time PCR to detect the viral load. The detailed protocol was described as Wu et al. (2017).

### Histopathological Examinations

The tissue samples were dehydrated and embedded in paraffin wax, and serial paraffin sections (4 μm) were obtained. Shortly, the sections were immersed in three consecutive 5 min xylene washes to remove paraffin and were subsequently hydrated with five consecutive ethanol washes in descending order of concentration: 100, 95, 80, 70%, and deionized water. The paraffin sections were then stained with hematoxylin-eosin (H&E), and histopathological changes were visualized using a light microscope (LM, BX51, Olympus Co., Japan).

### Immunohistochemical Staining

Tissue sections of brain and spinal cord were prepared as described above. Immunohistochemical staining was performed using a HistostainTM-Plus kit following the manufacturer's instruction (ZSGB-BIO, Beijing, China) and then applied with 3,3′-Diaminobenzidine tetrahydrochloride (DAB, ZSGB-BIO, Beijing, China) to visualize the antigen-antibody compound, and counterstained with haematoxylin. The slides were visualized using a light microscope (LM, BX51, Olympus Co., Japan).

### Transmission Electron Microscope (TEM)

Brain and spinal cord samples were cut into pieces and fixed in 2.5% (v/v) glutaraldehyde-polyoxymethylene solution overnight at 4°C. The tissues were postfixed in 2% osmium tetroxide for 1 h at 4°C and embedded in araldite CY212 after dehydration in ascending grades of ethanol. Ultrathin sections (50–60 nm) were sliced and stained with alkaline and lead citrate uranyl acetate. The sections were examined under a JEM 1230 transmission electron microscope.

### Cell Culture

Primary human brain microvascular endothelial cells (HBMVECs) (BK-PM-010) were purchased from a company (Biopike Technology Company Ltd., Beijing, China) and cultured as described previously (Renou et al., 2014a). Briefly, cells were inoculated with HEV (300 multiplicity of infection) and collected in 48 h for immunofluorescence staining or western blotting. HEV-negative homogenate served as control. The data shown in immunofluorescence staining images were from one typical experiment out of three independent experiments and western blot below represented at least 3 experiments with similar results.

### Immunofluorescence Staining

Cells were fixed in 4% formaldehyde and permeabilized in 0.2% Triton X-100/PBS. After washing and blocking with 2% bovine serum albumin, the sections were incubated with antibodies for laudin5 (1:200; Boster Biological Technology Co., Ltd., China) or Vementin (1:200; Beijing Bioss Biological Technology Co., Ltd., China) overnight at 4°C. After washing, the appropriate secondary antibodies were applied (1:2000; CoWin Biosciences, China) and the nucleus were stained with DAPI. The images were analyzed by Fluorescence microscopy.

### Western Blotting

The protein concentration from HEV-treated human cerebral microvascular endothelial cells was determined with the BCA protein assay kit (Thermo Fisher Scientific, Waltham, MA, USA) and 30 μg of protein was separated by SDS-PAGE. The blots were incubated with anti-Claudin5 (1:600) and ZO-1 (1:500) purchased from Boster Biological Technology Co., Ltd. (China), Occludin (1:500) and VE-cadherin (1:500) from Beijing Bioss Biological Technology Co., Ltd. (China), and anti-β-tubulin (1:600; Boster Biological Technology Co., Ltd., Wuhan, China) as internal control for 16 h at 4°C. The membranes were then incubated with the secondary antibodies conjugated with horseradish peroxidase (Santa Cruz Biotechnology) and developed by enhanced chemiluminescence (Thermo Scientific). The images were analyzed with ImageJ (NIH, Bethesda, MD, USA) and data were normalized to the expression of anti-β-tubulin antibody.

### Statistical Analysis

The data shown represent at least 3 experiments and were analyzed by the 2-tailed, unpaired Student's *t*-test between 2 groups. The results are expressed as the mean ± SEM. *P* < 0.05 was considered statistically significant.

## Results

### Evaluation of Positive- and Negative-Strand of HEV RNA in Brain and Spinal Cord

To evaluate the virus infection and replication, HEV RNA in brain and spinal cord tissue of infected rabbits was detected by RT-nPCR at 7, 28, 48 dpi after intraperitoneal injection and 49 dpi, 63 dpi after gavage, and results were summarized in Table 1. The results showed that all the brain and spinal cord from control rabbits were tested negative for HEV RNA. Two rabbits infected via intraperitoneal injection were detected HEV RNA positive in the brains at 28 dpi, with viral load 2.90E2 and 1.18E2 copies/μl, respectively. One rabbit was detected HEV RNA positive in spinal cord at 28 dpi in intraperitoneal injection group, with viral load 4.35E2 copies/μl. Rabbits infected HEV via gavage were not tested positive at 49 and 63 dpi.

**Table 1:** Viral load detection in brain and spinal cord of experimentally infected rabbits.

**Sample/dpi**	**Via intraperitoneal injection**			**Via gavage**	
	**7 dpi**	**28 dpi**	**49 dpi**	**49 dpi**	**63 dpi**
HEV-positive brain No.	–	28C, 28D^a^	–	–	–
Viral load in brain	–	2.90E2, 1.18E2	–	–	–
HEV-positive spinal cord No.	–	28C^b^	–	–	–
Viral load in spinal cord	–	4.35E2	–	–	–

*DPI, days postinoculation. -, positive-strand and negative-strand of HEV RNA negative by RT-nPCR*.

a *brain positive-strand and negative-strand HEV RNA-positive rabbit serial number by RT-nPCR*.

b *spinal cord positive-strand and negative-strand HEV RNA-positive rabbit serial number by RT-nPCR*.

*HEV viral load in brain and spinal cord are detected by qPCR and shown as copies/μl*.

### Histopathological Lesions of Brain and Spinal Cord of Rabbits Experimentally Infected by HEV

**Figure 1:**
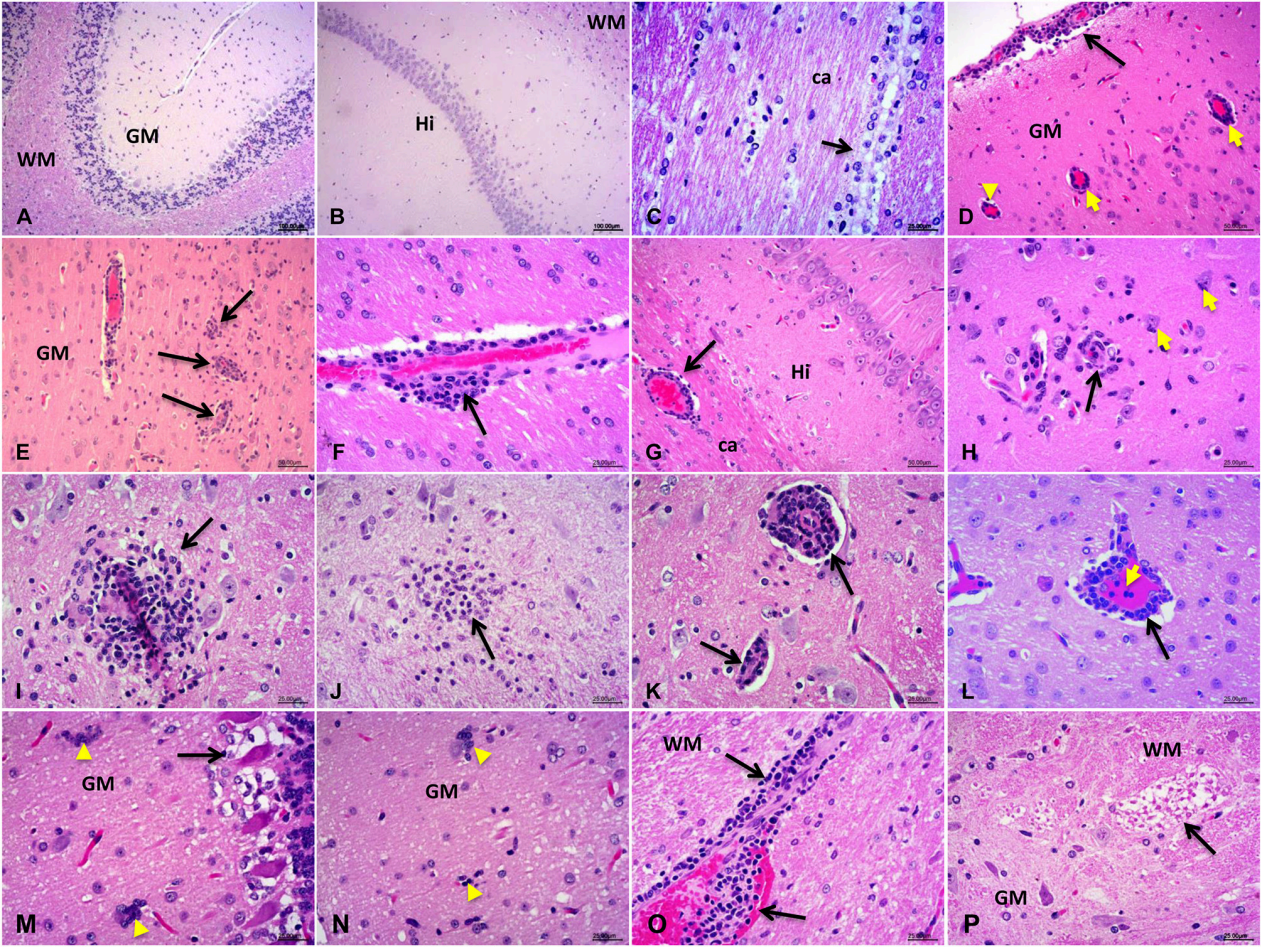
Histopathological detection of the central nervous system (CNS). **(A–C)** The normal structure of the CNS tissue from control rabbits: cerebellum **(A)** the molecular layer and granular layers of the gray matter (GM), and pukinje cells and the white matter (WM); cerebrum **(B)** hippocampus (Hi) and **(C)** corpus callosum (ca) and the ependymal epithelium of the white matter **(D–P)**. Various pathological changes observed in HEV-RNA positive brain sections, especially the characteristics of viral encephalitis include perivascular cuffs of lymphocytes and microglial nodules: cerebrum **(D–I)**: venous congestion of the Pia Mater, perivascular cuffs of lymphocytes and microglial hyperplasia in the cerebral cortex, corpus callosum (ca), brainstem, hypothalamus and the pons; cerebellum **(M)** degenerate and necrotic pukinje cells, **(N)** microglial nodules, and necrotic neurons surrounded and invaded by hypertrophic microglial cells (neuronophagia), **(O)** perivascular cuffs of lymphocytes, and hemorrhage; spinal cord **(P)**: degeneration of neuron axons of the white matter (demyelination).

The results obtained by H&E staining showed typical viral encephalitis changes in HEV-RNA positive rabbits, including meningoencephalitis, perivascular cuffs of lymphocytes and microglial nodules in the cerebrum and cerebellum tissues in rabbits experimentally infected by HEV (Figures 1D–L). Degradation and necrosis of neurons and Pukinje cells were found in the cerebellum sections of the infected rabbits (Figures 1M,N). Neuron axons degeneration (demyelination) of the white matter in spinal cord of experimental rabbits was examined (Figure 1P). Additionally, vascular congestion and hemorrhage occurred commonly in brain and spinal cord of the experimental rabbits. No obvious injuries were observed in control rabbits (Figures 1A–C).

### Immunohistochemistry Staining for HEV ORF2 Antigen in Brain and Spinal Cord

**Figure 2:**
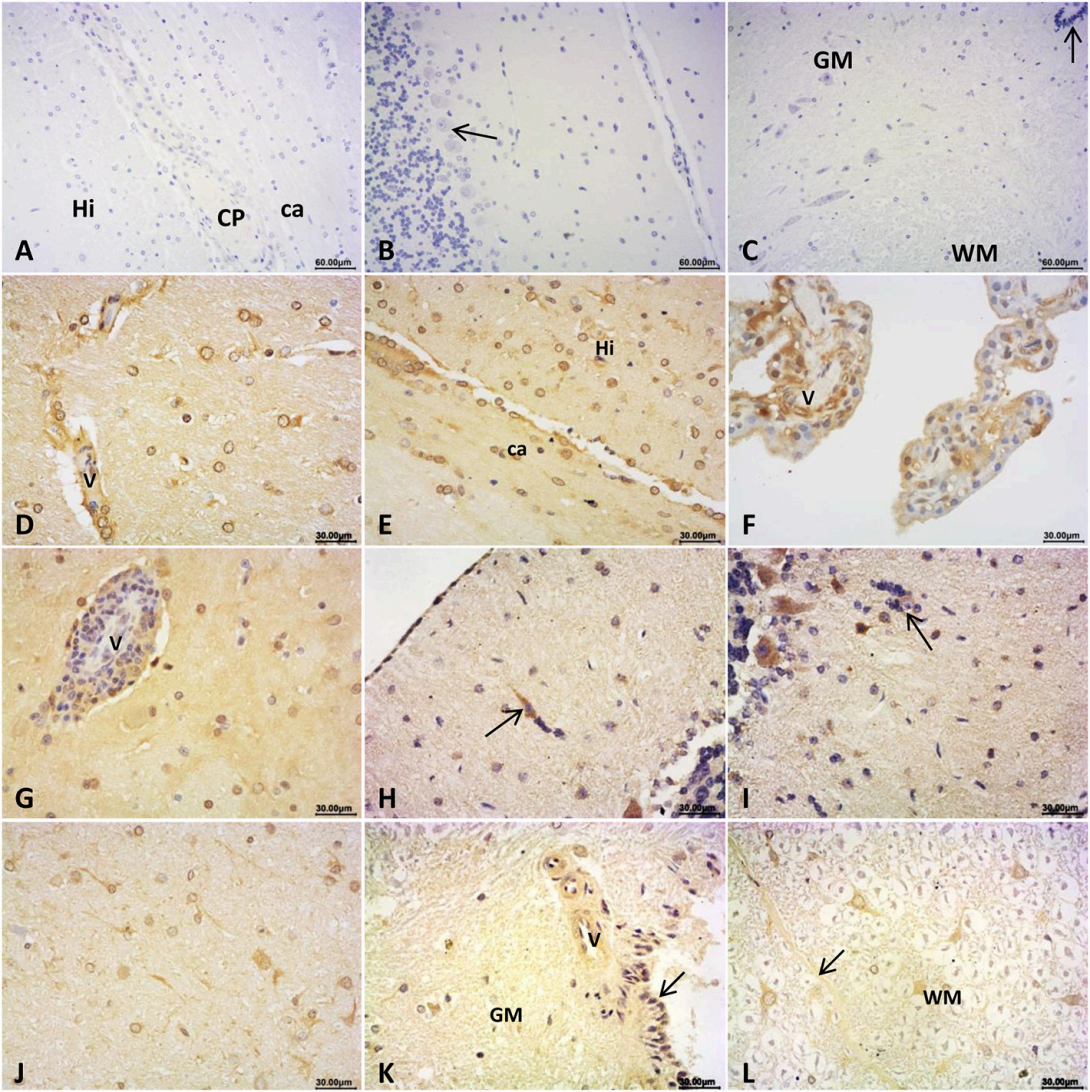
Immunohistochemical staining for HEV ORF2 antigen. **(A–C)** ORF2 positive signals was not detected in brain and spinal cord of the control rabbits: cerebrum **(A)** hippocampus and white matter area (Hi: hippocampus, CP: choroid plexus, ca: corpus callosum), cerebellum **(B)** the molecular layer and granular layers, and pukinje cells, spinal cord **(C)** the gray matter (GM) and white matter (WM), arrow showed central canal of the spinal cord. In brain detected as HEV RNA positive, HEV ORF2 protein expression distributed at: **(D–G)** cerebrum, **(D)** perivascular area and cytoplasm of glial cells of the white gray, **(E)** cytoplasm of glial cells in corpus callosum (ca) and hippocampus (Hi) of the white matter, **(F)** cytoplasm of choroid epithelium cells, and **(G)** lymphocytes in perivascular area and glial cells cytoplasm of the gray matter; **(H–I)** cerebellum, **(H)** Pia Mater and neurons invaded by microglial cells, **(I)** cytoplasm of pukinje cells and cells that formed microglial nodules (arrow); **(J)** cytoplasm of neurons and glial cells in white matter of pons; **(K,L)** spinal cord, **(K)** perivascular area and epithelial cells of central canal of the gray matter, **(L)** posterior septum (arrow) of spinal cord and cytoplasm of neurons in white matter (WM).

To figure out the expression of HEV antigen in brain and spinal cords, IHC staining was conducted for HEV ORF2 protein. ORF2 antigen was detected in brain and spinal cord in rabbits detected as HEV-positive. Positive signals distributed mainly in cytoplasm of neuroglial cells and choroid epithelium cells in cerebrum (Figures 2D–G); and in pia mater and injured neurons, Pukinje cells in cerebellum (Figures 2H,I); and in neurons of the pons (Figure 2J). In the spinal cord, HEV ORF2 protein was visible in cytoplasm of neurons (Figures 2K,L). No positive signal was observed in the CNS from control rabbits (Figures 2A–C).

### Ultrastructure of BBB Injury After HEV Infection

**Figure 3:**
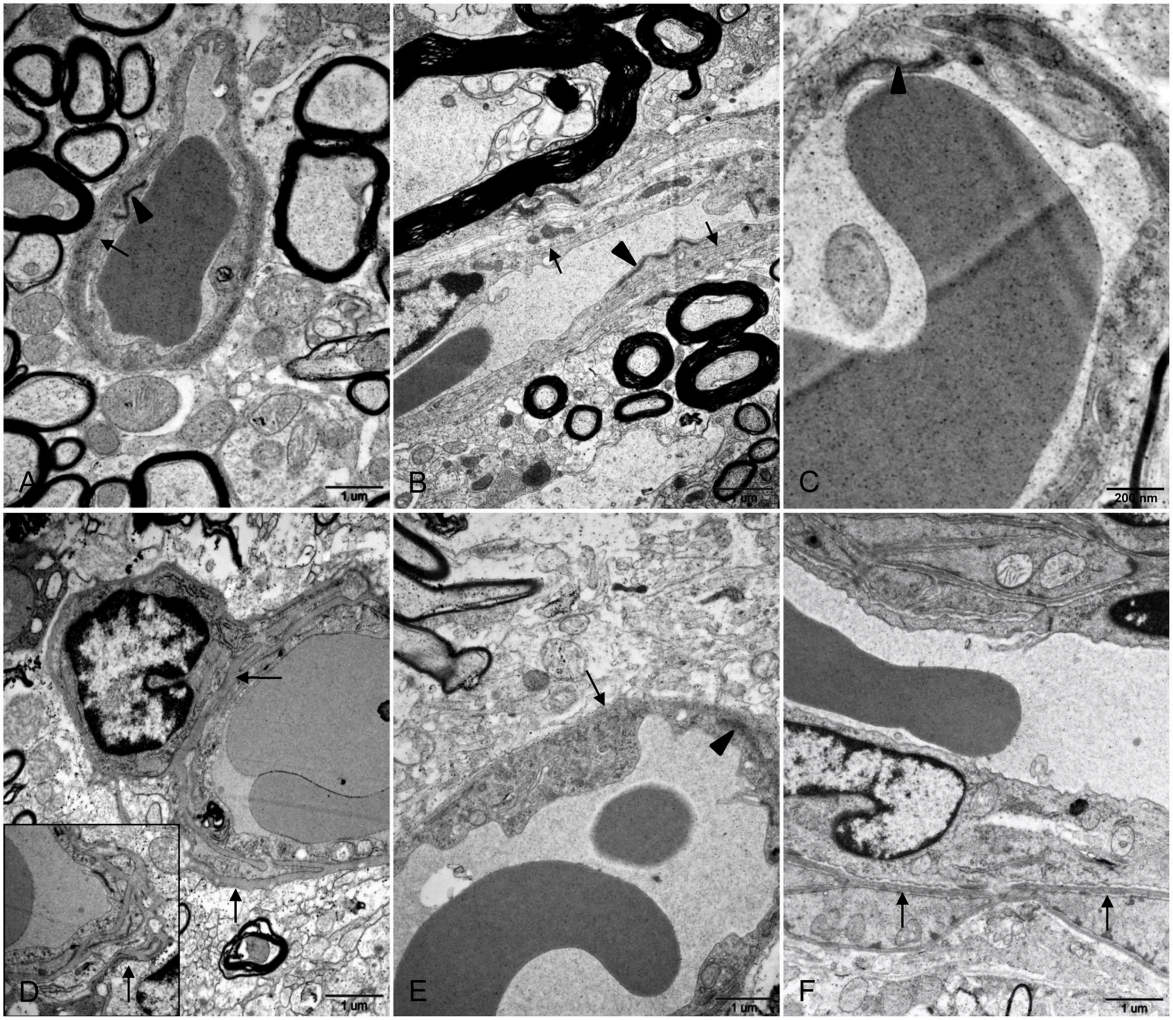
Ultrastructural examination of BBB in brain and spinal cord tissues. **(A–C)** Basement membranes of blood vessel wall was intact and smooth (arrows) and junctional complexes between the endothelium were compact and clearly defined (arrowheads) in control brain **(A)** and spinal cord **(B,C)** tissues. **(D–F)** In brain tissues from HEV RNA-positive gerbils, basement membranes of capillary endothelium as well as pericytes was thickened and reduplicated (arrows) **(D)**. In spinal cord tissues positive with HEV RNA, basement membranes of blood vessel endothelium was irregular and thickened (arrows) **(E,F)**, and junctional complexes between endothelial cells were indistinct (arrowhead) **(E)**.

Transmission electron microscope (TEM) was used to determine morphological injury of BBB. No apparent lesions were observed in brain and spinal cord tissues of control gerbils. Basement membranes of capillary endothelium were smooth and continuous with uniform thickness and junctional complexes between the endothelium were compact and clearly defined (Figures 3A–C). In HEV RNA-positive tissues from the experiment group, disruption of BBB was determined, including irregular and thickened basement membranes and indistinct junctional complexes between endothelial cells of spinal cord tissues (Figures 3E,F). In brain tissues with HEV RNA, it was found that basement membranes of endothelium and surrounded pericytes were thickened and reduplicated (Figure 3D).

### Tight Junction Related Proteins Claudin5, Occludin, and ZO-1 Decreased in HBMVECs Experimentally Infected With HEV

**Figure 4:**
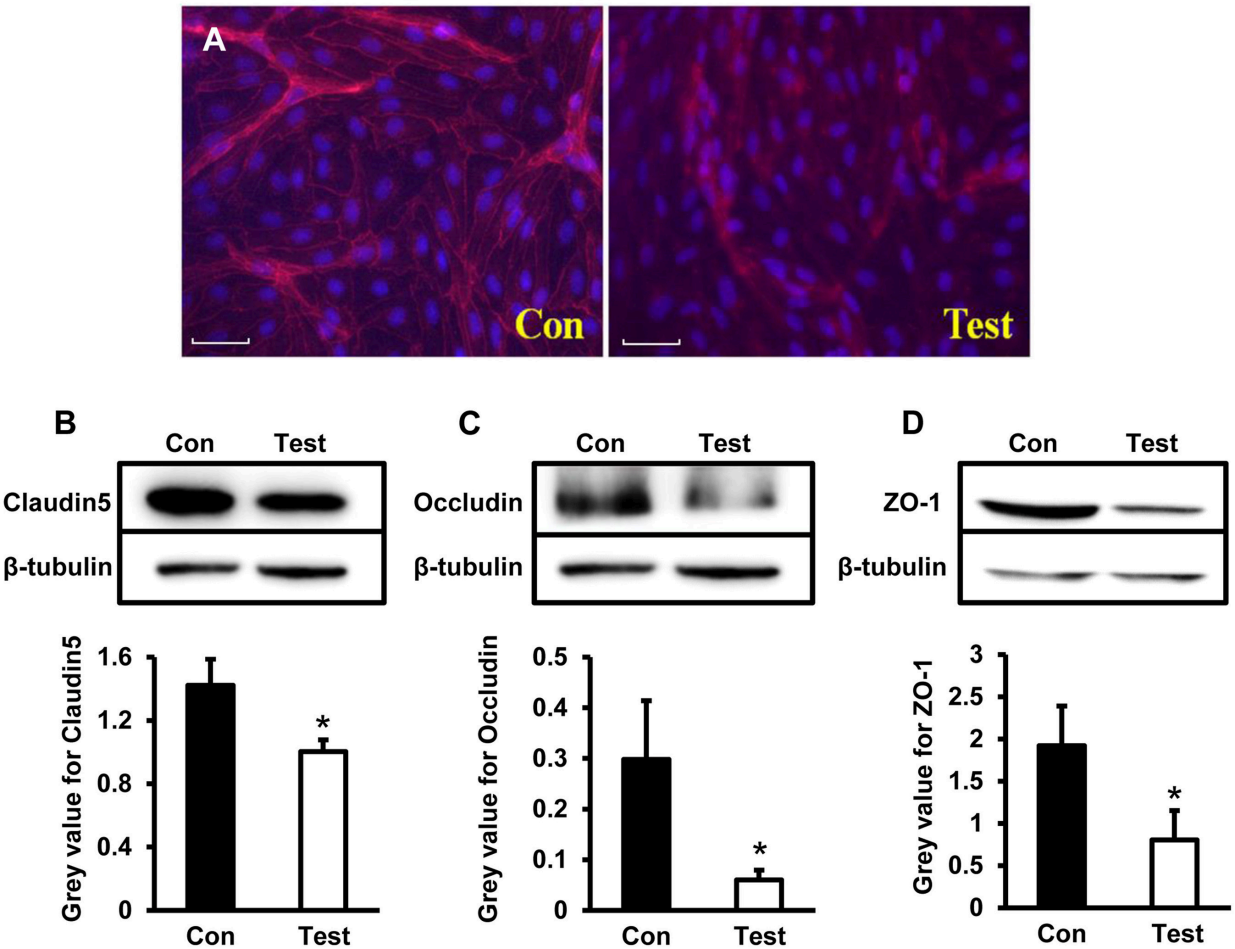
Quantity analysis of tight junction-associated proteins (Claudin5, Occludin, ZO-1) in HBMVECs. **(A)** Representative images of Immunofluorescence staining. The images showed that positive signals of Claudin5 distributed consecutively in membrane of control cells, and expression levels decreased after HEV infection and positive signals translocated into cell cytoplasm (Red: Claudin, Blue: DAPI). **(B)** Western blot indicated that expression levels of Claudin5 were significantly reduced in HEV-infected cells compared to control group (^*^P < 0.05). **(C,D)** Deceased protein levels of Occludin and ZO-1 were also detected in HEV-infected cells (^*^P < 0.05).

To further explore expression of tight junction proteins during HEV infection, Claudin5, Occludin, and ZO-1 were examined by both immunofluorescence staining and western blot in HBMVECs infected with HEV. Under the microscopy, expression of Claudin5 in HBMVECs were consecutively located in membrane of the cells in control group; Claudin5 positive signals in HEV-infected cells decreased and aggregated in cell cytoplasm (Figure 4A). Western blot showed expression levels of Claudin5 were significantly decreased in experimental cells compared to control (*P* < 0.05) (Figure 4B). Levels of Occludin and ZO-1 were also reduced in HEV-treated cells (*P* < 0.05) (Figures 4C,D).

### Adhesion Junction Related Protein VE-Cadherin Increased in HEV Infected HBMVECs

**Figure 5:**
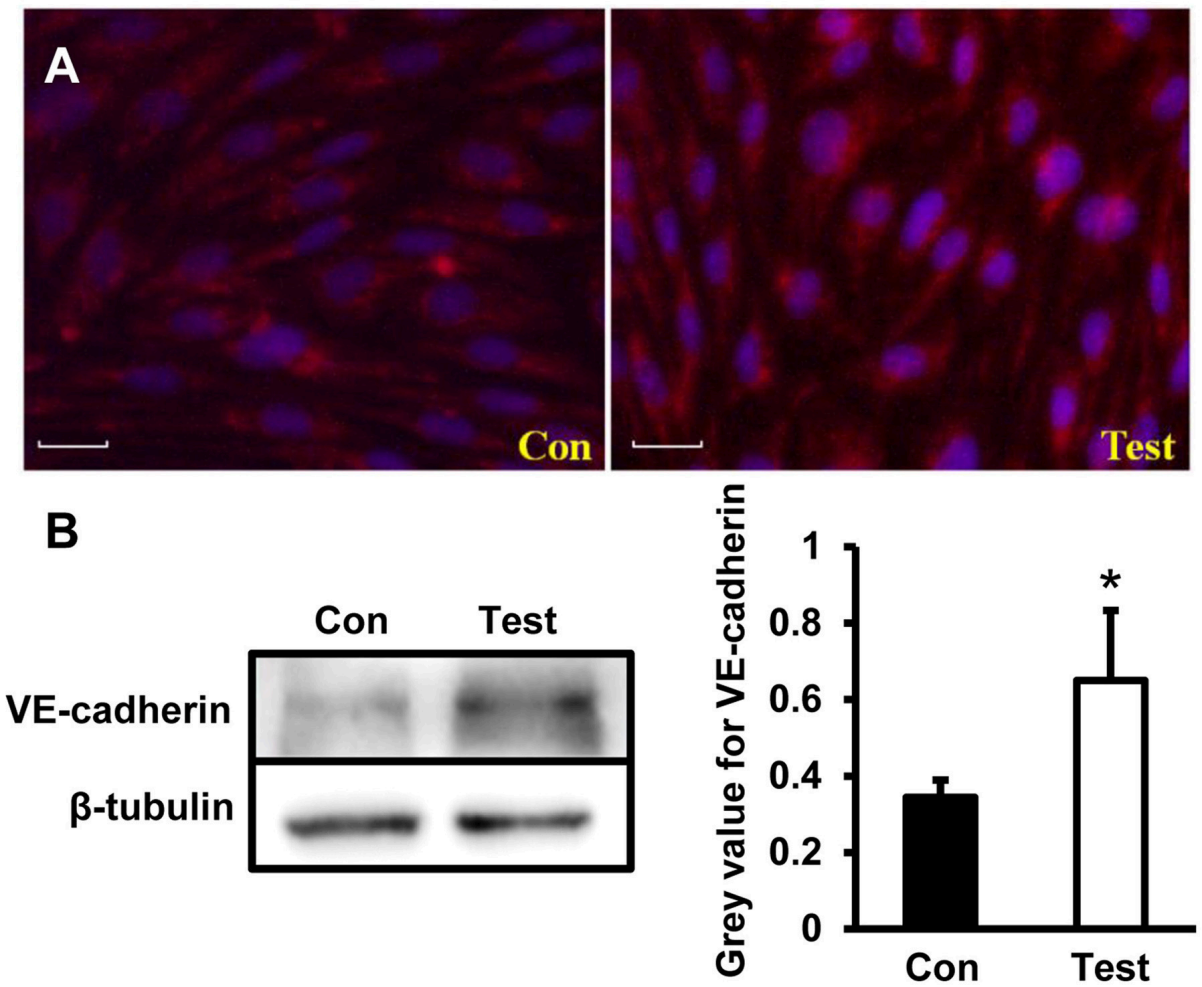
Quantity analysis of adherent junction protein (VE-cadherin) in HBMVECs. **(A)** Representative images of Immunofluorescence staining. The images showed that VE-cadherin expressed as scattering points around cell membrane between two adjacent cells in control cells, and positive signals increased in HEV-infected cells (Red: VE-cadherin, Blue: DAPI). **(B)** Western blot analysis showed that VE-cadherin expression levels were increased in HEV-infected HBMVECs (^*^P < 0.05).

To further investigate changes of adhesion junction protein during HEV infection, VE-cadherin were tested in HBMVECs infected with HEV. Under the microscopy, VE-cadherin expressed as scattering points along cell membrane between two adjacent cells in control group; expression levels of VE-cadherin increased in HEV-infected cells compared to control group (Figure 5A). Western blot showed increased protein expression of VE-cadherin in HEV-infected HBMVECs (*P* < 0.05) (Figure 5B).

### Expression of Vimentin Did Not Change Significantly in HEV Infected HBMVECs

**Figure 6:**
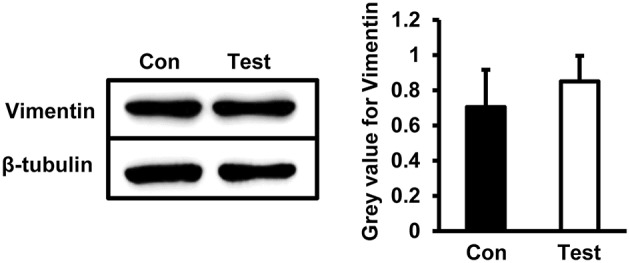
Quantity analysis of Vimentin in HBMVECs. Western blot analysis showed that protein levels of Vimentin was not significantly changed in HEV-infected cells.

To determine whether HEV infection alters cell stability, a cytoskeleton protein Vimentin was examined in HBMVECs infected with HEV. Western blot showed no significant change of Vimentin expression in cells infected with HEV (Figure 6).

## Discussion

A number of studies have reported neurological manifestations in HEV-infected patients or laboratory animals, with evidence of HEV replication in neuronal cells (Pischke et al., 2017). It was found that Guillain-Barré syndrome with abnormal alanine aminotransferase (ALT) levels usually associated with HEV infection (Stevens et al., 2017). Our previous data showed that ALT level was significantly increased in the experimentally infected rabbits at 28 and 49 dpi, and HEV RNA was detected in multiple tissues, including the liver, salivary gland, tonsil, spleen, lymph nodes, small intestine, Sacculus Rotundus (SR), cecum, and appendices (Wu et al., 2017). In the present study, HEV RNA (positive and negative strand) was tested in brain (2/4) and spinal cord (1/4) at 28 dpi in experimental rabbits inoculated with HEV via intraperitoneal injection. These results further confirm that rabbits is susceptible to HEV under experimental conditions, with a risk of CNS infection.

To date, very limited pathological injuries were documented in clinical and laboratory studies of HEV infection, though a number of studies have reported that HEV infection associated with clinical syndromes such as Guillain-Barré syndrome, neuralgic amyotrophy, and encephalitis/meningoencephalitis/myositis (Dalton et al., 2016). In gerbils experimentally infected HEV, pathological changes, including neurons degeneration and necrosis, neuronophagia, microglia nodules, inflammatory cells infiltrating to the ependymal epithelium, choroid plexus region hemorrhage and inflammatory cells infiltration, “perivascular cuff” formation, Purkinje cells necrosis, hemorrhage in dorsal median septum and central canal of spinal cord were observed (Shi et al., 2016). These pathological changes were in accordance with clinical manifestations in HEV infected patients. Rabbits are considered to be useful model to explore the pathogenesis of hepatitis E, especially the extrahepatic injury of HEV (Nimgaonkar et al., 2018). In our study, pathological changes particularly viral encephalitis associated perivascular cuffs of lymphocytes and microglial nodules were observed in rabbits infected with HEV. Together with neuron injuries detected, our results indicated that HEV might directly involve in the CNS disorders.

There are 3 opening reading frames (ORF) of HEV, including the viral replicase (belongs to ORF1), the viral capsid protein (ORF2), and a small phosphoprotein might involve in the secretion of viral particles (ORF3) (Debing et al., 2016). However, a recent study showed that only ORF2 was unequivocally detectable in FFPE liver samples with hepatitis E, and the distribution pattern of HEV ORF2 particles was not only in cytoplasm and bile canaliculi but also in nucleus of hepatocytes (Lenggenhager et al., 2017). Most importantly, they found that HEV ORF2 expression correlated with the detection of HEV RNA *in situ* by ISC, and IHC for ORF2 protein, indicating that ORF2 was sufficiently sensitive for HEV detection in a period of high viral load (Lenggenhager et al., 2017). Interestingly, HEV ORF2 protein was observed in the cerebellum granule cell layer in brain tissue of infected mice and chronically infected monkeys (Zhou et al., 2017). *In vitro* experiments further revealed that both primary cerebellar and hippocampal neurons of mice were susceptible to HEV infection and capable of producing the viral ORF2 protein (Zhou et al., 2017). In our study, HEV ORF2 particles were detected in cytoplasm or nucleus of cells in brain and spinal cord tissues of the HEV RNA positive rabbits, such as glial cells, microglial cells, choroid epithelium cells, and neural cells, especially in cells located in perivascular areas. These results suggested that perivascular cells and neural cells are targets of HEV in CNS, which is in accordance with our previous study in gerbils (Shi et al., 2016). In gerbils experimentally infected with HEV, neurocytes degeneration and necrosis, particularly the blood vessel wall components of the brain and spinal cord sections, including swelling endothelium and loss of endothelial junctional complexes, were observed using a transmission electron microscopy (Shi et al., 2016). We also found that basement membranes of the blood vessel was thickened and even reduplicated in brain and spinal cord tissues of HEV infected gerbils, which might be a compensatory response to BBB disruption. Together, these findings suggested that HEV infection result in a leakage of the BBB, which facilitated the virus transmission into the central nervous tissue.

The BBB is comprised of highly specialized and tightly sealed monolayer of brain microvascular endothelial cells (BMVECs), and other immune and neural cells, with elaborate junctional complex (both tight junctions and adherens junctions such as ZO-1, Claudins, Occludin, VE-cadherin) and integrated basal lamina which restricted the entry of pathogens and other soluble molecules into the CNS (Bernas et al., 2010; Miner and Diamond, 2016). Junctional proteins were reported to play critical roles during neurological disorders such as experimental autoimmune encephalomyelitis (EAE), Alzheimer's disease and Parkinson's disease (Pfeiffer et al., 2011; Cabezas et al., 2014; Halliday et al., 2016). Besides, neurotropic viruses such as West Nile virus, Japanese encephalitis virus (JEV) and Rabies virus infection are causes of BBB disruption which in turn facilitate the spread of virus into brain parenchyma from blood circulation. Loss of ZO-1 was detected in HBMVE cells incubated with supernatant media from WNV-infected HBCA cells (Verma et al., 2010). Occludin expression was downregulated by CXCL10 in bEnd.3 cells co-cultured with brain extracts infected with RABV (Chai et al., 2015). Expression of tight junction proteins occludin, claudin-5 and ZO-1 in JEV-infected mice brain were overall reduced (Li F. et al., 2015). The above study indicated that directly crossing the BBB is one of the most common pathways of viruses invading the CNS, and breaking the junctional complexes integrity will be a key factor. In the present study, primary HBMVECs were employed to evaluate roles of tight junctions and adherens junctions during HEV infection. We found that expression of tight junction proteins Claudin5, Occludin, and ZO-1 decreased in HBMVECs treated with HEV, indicating a direct invasion of HEV into the CNS via disruption of tight junction complex of HBMVECs. However, further investigation showed that VE-cadherin, an adherens junction protein participating into the cohesion and organization of the intercellular junctions of endothelial cells, was upregulated in HBMVECs infected with HEV. It might be a compensatory response to dysfunction of tight junction during HEV invasion. The cytoskeleton protein Vimentin was not changed in the infected cells, indicating that HBMVECs stability might not be largely affected by HEV infection.

**Figure 7:**
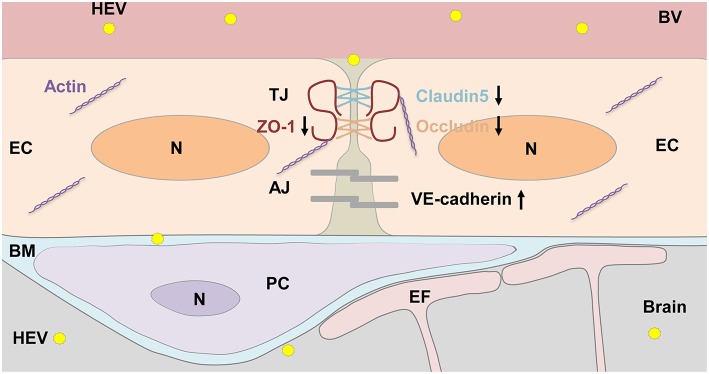
HEV entry to the brain. Components of the BBB including tight junction (TJ) and adhesion junction (AJ) between endothelial cells (EC), pericytes (PC), astrocytes endfoot (EF), as well as basement membrane (BM) surrounded ECs and PCs were shown in the graph. HEV infection decreased expression levels of TJ proteins, including ZO-1, Occludin, and Claudin5, increased AJ protein VE-cadherin expression, leading disorder of junctional complexes between capillary ECs, further facilitating HEV invasion into the brain tissue. In responding, basement membrane of PCs and ECs became thickened to protect brain tissue from HEV infection. N, Nucleus; BV, blood vessel.

In summary, the BBB of brain is a potential target of HEV invasion into the CNS in experimentally infected rabbits (Figure 7). Further study should be focused on roles of tight junction proteins such as Claudin5, Occludin, and ZO-1 in integrity of BBB during HEV infection. It will help understand pathogenesis of the CNS injury caused by HEV and seek potential therapeutics for HEV infection in the future.

## Author Contributions

JT, RShi, and RShe performed the study concept and design. RShi, TLiu, QW, and JA performed the laboratory work and data analysis. WH and MS performed the analysis and interpretation of data. JT and RShi wrote the paper. All of the authors read and approved the final article.

### Conflict of Interest Statement

The authors declare that the research was conducted in the absence of any commercial or financial relationships that could be construed as a potential conflict of interest.
